# Silicic Acid Removal by Metal-Organic Frameworks for Silica-Scale Mitigation in Reverse Osmosis

**DOI:** 10.3390/membranes13010078

**Published:** 2023-01-08

**Authors:** Rui Guo, Jun Zhang, Taona Nashel Mufanebadza, Xinxia Tian, Lixin Xie, Song Zhao

**Affiliations:** 1 Chemical Engineering Research Center, School of Chemical Engineering and Technology, Tianjin University, Tianjin 300072, China; 2 Tianjin Key Laboratory of Membrane Science and Desalination Technology, State Key Laboratory of Chemical Engineering, Tianjin University, Tianjin 300072, China; 3 Institute of Seawater Desalination and Multipurpose Utilization, MNR (Tianjin), Tianjin 300192, China

**Keywords:** MIL-101(Fe) MOF, adsorption, reverse osmosis, pretreatment, silicic acid removal

## Abstract

Reverse osmosis (RO) membranes are susceptible to silica scaling, resulting in irreversible degradation of membrane performance. This work covered the fabrication of MIL-101(Fe) for silicic acid adsorption to alleviate the silica scaling of RO membranes. The effect of pH, mixing time and initial concentration on silicic acid adsorption of MIL-101(Fe) was appraised in detail. The adsorption experiments demonstrated that MIL-101(Fe) possessed an excellent adsorption ability for silicic acid with the maximum adsorption capacity reaching 220.1 mgSiO_2_·g^−1^. Data fitting confirmed the pseudo-second-order equation and Freundlich equation were consistent with silicic acid adsorption on MIL-101(Fe). Finally, a simulated anti-scaling experiment was carried out using a feed solution pretreated by MIL-101(Fe) adsorption, and the permeance exhibited a much lower decline after 24 h filtration, confirming that MIL-101(Fe) exhibits an excellent application potential for silica-scale mitigation in RO systems.

## 1. Introduction

Water resource shortages have become an international problem worthy of concern in industrial production and daily life [1,2], seriously affecting household living quality and global sustainable development [3,4]. To solve this problem, RO systems have been widely used due to their ease of operation, efficient water treatment and low environmental pollution [5,6,7]. However, inorganic scaling is a commonly encountered bottleneck for high water recovery of RO systems, leading to a reduction in flux and extra cost of energy consumption and operation.

Inorganic pollution generally results from the deposition of salts on the surface or in the pores of membranes when the ion concentration exceeds its solubility product. Salts with low solubility, including calcium carbonate (CaCO_3_) [8,9], calcium sulfate (CaSO_4_) [10,11] and sodium metasilica (Na_2_SiO_3_) [12,13], are the most common inorganic scales on the membrane surface. Among them, silica scale is worthy of more attention because of the high concentration of SiO_2_ in water (20–60 mg·L^−1^) [14]. The formation mechanism of silica scale has been researched, and it was confirmed that the interaction between silicic acid and the membrane surface had a decisive effect on the formation rate of scale [15,16]. Kempter et al. surveyed the formation process of silica scales with atomic force microscopy (AFM). After the membrane was immersed in a Na_2_SiO_3_ solution (250 mg/L) for 1 h, SiO_2_ particulates with a diameter of 20–30 nm began to appear, and the membrane surface was completely covered with SiO_2_ after 9 h of immersion [17].

During the actual operation of an RO device, cleaning-in-place is generally applied to remove fouling [18]. Acidic cleaning can dissolve inorganic scales, and alkaline cleaning can remove organic fouling and biofouling [19]. However, silica scale generated by the polymerization of silicic acid is hard to remove with conventional acidic cleaning [20]. Only ammonium bifluoride (NH_4_HF_2_) and hydrofluoric acid (HF) are effective against silica scale [21], but they may cause serious damage to polymeric membranes and RO plants, resulting in a reduction in salt rejection [22]. Fortunately, highly alkaline solutions could promote the depolymerization of colloidal silica, leading to an increase in the solubility of silica [23]. However, alkaline cleaning generally takes considerable time and cannot completely dissolve silica scale. Thus, it is urgent to develop effective approaches to prevent silica scale in RO systems.

Four approaches can be employed to prevent silica scale in RO systems: the pretreatment of feed solution, the addition of antiscalant, the optimization of operational parameters and the functional modification of membrane materials [24,25]. The latter three methods have shortcomings. For instance, scale inhibitors cannot completely prevent the formation of scale [26], and may cause more serious chemical pollution. The optimization of operational parameters would significantly increase energy consumption [27], and the functional modification of membrane materials is still in the experimental stage. Comparatively, the pretreatment of feed solutions has been widely used in RO systems due to its ease of operation, stability and low capital cost. Compared to coagulation, electrocoagulation and ion exchange, adsorption produces little solid sludge and insoluble metal silicate scale [18], and suffers little from other anionic species [28], making it one of the most effective pretreatments. Guan et al. proposed the synthesis of nanomaterials with a shell of Al(OH)_3_ that provided the ability to adsorb silica and a core of superparamagnetic Fe_3_O_4_ that enabled the magnetic recovery. It was reported that the Al(OH)_3_@Fe_3_O_4_ (2 g·L^−1^) could remove more than 80% of silicic acid from 2 mM initial silicic acid concentrations [29]. Naren et al. investigated the adsorption kinetics of silicic acid on akaganeite under different pH values [30]. Chloride ions bound to the akaganeite surface were liberated and then Fe-OH or Fe-O^−^ bonds formed, which afterwards acted as unsaturated ion sites for silicic acid adsorption. These above reports highlighted the promise of nanomaterials for treating brackish water and realizing a superior desalinated water recovery.

MOFs are compounded by metal ions or metal ion clusters and organic linkers, and demonstrate structured crystal lattices with a specific pore size distribution (PSD) [31,32]. Compared with other porous materials, MOFs have advantageous features, including diversity [33], high porosity [34], enormous surface area [35], uniformly distributed metal sites [36,37], mild synthesis conditions and variability in physicochemical properties [38,39]. Owing to their enormous surface area and adjustable porosity, MOFs have been extensively investigated for gas or ionic adsorption [40,41,42]. Zhao et al. demonstrated that Fe-MIL-101 with unsaturated ion sites allowed the selective and irreversible adsorption of SeO_3_^2−^. This adsorption process showed an adsorption capacity of 183.7 mgSeO_3_^2−^·g^−1^ and rapid kinetics, resulting from the large pore size and numerous unsaturated ion sites of Fe-MIL-101 by forming Fe-O-Se bonds [43]. In the study of Ni et al., three kinds of MOFs were synthesized to adsorb silicic acid from wastewater [44]. Al, Fe and Cu were employed as metal precursors to synthesize MOFs with a maximum silicic acid adsorption capacity of 201.4 mgSiO_2_/g for MOF-Cu. Thus, the excellent performance of MOFs for silicic acid adsorption shows fantastic potential applications for silica-scale mitigation for RO membranes.

Herein, to alleviate the silica scaling of RO membranes, MIL-101(Fe) was prepared through the reaction of FeCl_3_·6H_2_O and P-phthalic acid (H_2_BDC) due to its adsorption potential of silicic acid [45]. The effect of pH value, mixing time and initial concentration on silicic acid adsorption of MIL-101(Fe) was appraised. The adsorption kinetics, isotherm and thermodynamics of MIL-101(Fe) for silicic acid were analyzed to reveal the adsorption mechanism. Furthermore, MIL-101(Fe) for silicic acid adsorption was applied for the pretreatment of the feed solution of RO systems in order to confirm its application potential for silica-scale mitigation in RO systems.

## 2. Materials and Methods

### 2.1. Materials

Sodium sulfite (Na_2_SO_3_, AR) was obtained from Tianjin Kermel Chemical Reagent Co., Ltd. *N*,*N*-Dimethylformamide (DMF, AR), FeCl_3_·6H_2_O (AR), sodium metasilica (Na_2_SiO_3_, 44~47% SiO_2_) and H_2_BDC (99%) were obtained from Aladdin Reagent Co., Ltd. (Shanghai, China). Ethanol absolute (C_2_H_6_O, ≥99.7%), potassium bromide (KBr, AR) and ammonium molybdate ((NH_4_)_2_MoO_4_, 99%) were purchased from Jiangtian Chemical Reagent Co., Ltd. (Tianjin, China). Oxalic acid (C_2_H_2_O_4_, 99%) and 1-Amino-2-naphthol-4-sulfonic acid (C_10_H_9_NO_4_S, 95%) were obtained from Tianjin Chemart Chemical Technology Co., Ltd. (Tianjin, China). Commercial RO membranes (FILMTEC^TM^ LC 4040) were provided by Dow Chemical Company. Deionized water with a conductivity less than 20 μS/cm was produced using a laboratory RO systems.

### 2.2. MIL-101(Fe) Fabrication and Characterization

MIL-101(Fe) was fabricated through a simple solvothermal method using FeCl_3_·6H_2_O and H_2_BDC as the monomers [43,46]. In the first step, FeCl_3_·6H_2_O (4 mmol, 1.08 g) and H_2_BDC (4 mmol, 0.665 g) were added into DMF (30 mL). After stirring at room temperature for 30 min, the mixture was transferred to an autoclave, heated at 110 °C for 20 h and then cooled naturally. Finally, the reddish-brown precipitate was washed using DMF and ethanol three times each and then dried at 80 °C under vacuum overnight.

The chemical composition of MIL-101(Fe) was analyzed using X-ray photoelectron spectroscopy (XPS, ESCALAB 250xi, Thermo Fisher Scientific, Waltham, MA, USA) and Fourier transform infrared spectroscopy (FTIR, IS-10, Thermo Nicolet Corporation, USA). The surface morphology was characterized with FE-SEM (Nova Nano430, Thermo Fisher Scientific, America). The crystalline structure of MOF particles was investigated using X-ray diffractometry (XRD, Miniflex 600, Rigaku Corporation, Tokyo, Japan). Nitrogen adsorption–desorption curves were acquired by N_2_ adsorption using a desorption analyzer (Auto-Sorb-iQa 3200-4, Quantatech International Corporation, USA) to analyze the PSD of MOF particles.

### 2.3. Evaluation of Adsorption Performance

An adsorption experiment was performed to investigate the silicic acid adsorption ability of MIL-101(Fe). First, 0.5 g·L^−1^ MIL-101(Fe) was mixed with 120 mg·L^−1^ silicic acid solution, and the solution was sampled periodically until adsorption equilibrium. Then, the silicic acid concentration could be obtained by querying the standard curve. The adsorption rate (*R*) was calculated with Equation (1).

(1) $$R=\frac{C_{0}-C_{e}}{C_{0}}\times 100\%$$

where *C*_0_ (mg·L^−1^) and *C_e_* (mg·L^−1^) correspond to silicic acid concentrations in the initial and equilibrium solutions, respectively. The instantaneous adsorption capacity (*q*) was calculated using Equation (2).

(2) $$q=\frac{\left(C_{0}-C\right)V}{W}$$

where *C* (mg·L^−1^) is the silicic acid concentration, *V* (L) is the volume of silicic acid solution and *W* (g) is the quality of MIL-101(Fe).

Silico-molybdic acid spectrophotometry was used to determine the concentration of silicic acid. Specifically, 2 mL of ammonium molybdate solution (0.1 g·mL^−1^) and 1 mL of 18 wt% HCl solution were added to 20 mL of diluted sample. The solution was mixed evenly and then allowed to stand for 5 min. Afterwards, 1.5 mL of oxalic acid solution (1 mol/L) was added and mixed again. One minute later, 2 mL C_10_H_9_NO_4_S was added and set aside for 10 min. Silicic acid was quantified using UV-Vis spectroscopy at 420 nm. The concentration could be obtained by querying the standard curve of silicic acid.

### 2.4. Membrane Silica-Scaling Experiment

The silica-scaling experiment was carried out utilizing a cross-flow filtration RO device. The feed solution for the experiment was prepared by mixing 2000 mg/L NaCl, 770 mg/L CaCl_2_, 500 mg/L MgCl_2_ and 340 mg/L Na_2_SiO_3_ (the concentration of silica was 167 mg/L) with a pH value of 6.5 ± 0.1. The feed solution with or without the pretreatment of MIL-101(Fe) was used to investigate the permeance decline of the RO membrane during 24 h of continuous filtration. The filtration experiment was conducted at 25 ± 1 °C and the initial flux was adjusted to ~62 L·m^−2^·h^−1^ by regulating the pressure.

## 3. Results and Discussion

### 3.1. Structural Morphology of MIL-101(Fe)

**Figure 1:**
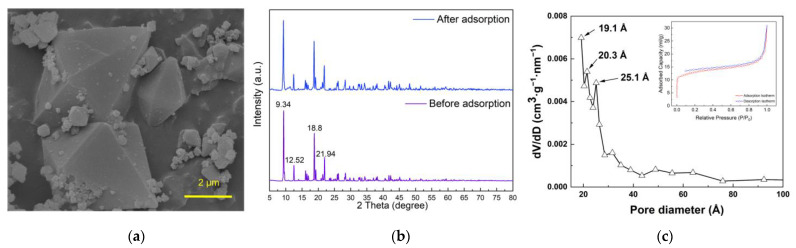
(**a**) SEM image of MIL-101(Fe); (**b**) X-ray diffraction curve of MIL-101(Fe) before and after adsorption; (**c**) PSD and BET isotherm of MIL-101(Fe).

As seen from the SEM image presented in Figure 1a, the synthesized MIL-101(Fe) presented a smooth octahedral structure, which was consistent with the structure reported previously [47,48]. The XRD curve of MIL-101(Fe) shown in Figure 1b exhibited intense peaks at 2θ values of 9.3°, 12.48°, 18.76° and 21.9°, corresponding to the previous reports [49,50], confirming the well-developed MIL-101(Fe) crystals. The PSD was characterized using the Brunauer–Emmett–Teller (BET) method and analyzed through density functional theory (DFT). It can be seen from Figure 1c that three narrow peaks are observed in the PSD profile (19.1 Å, 20.3 Å and 25.1 Å) [51], indicating the existence of micropores and mesopores in MIL-101(Fe).

### 3.2. Chemical Composition of MIL-101(Fe)

**Figure 2:**
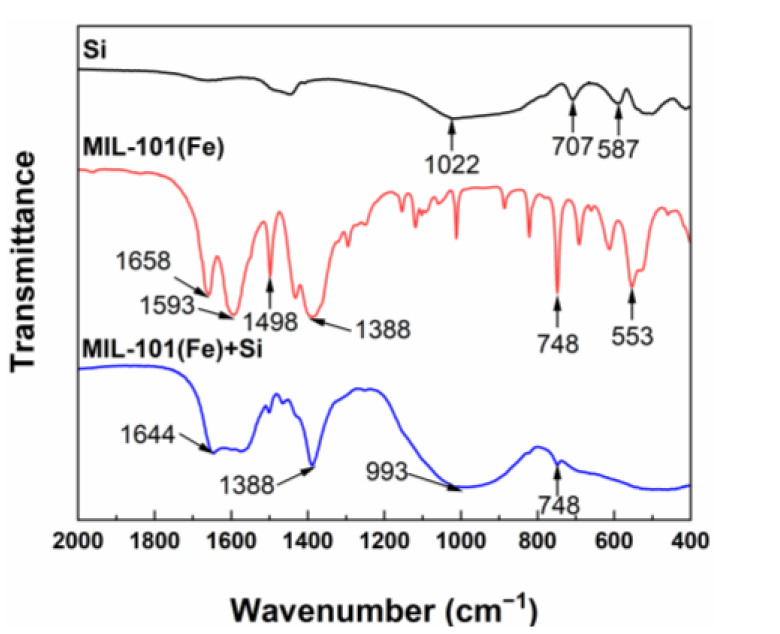
ATR-FTIR spectra of Si (black), MIL-101(Fe) before adsorption (red) and MIL-101(Fe) after adsorbing silicic acid (blue).

The chemical compositions of MIL-101(Fe) and sodium metasilica were determined using ATR-FTIR analysis. It can be seen from Figure 2 that the characteristic peaks of MIL-101(Fe) included carboxyl groups (1658 cm^−1^, 1593 cm^−1^ and 1388 cm^−1^), benzene groups (1498 cm^−1^), and chelate bonds formed by Fe^3+^ with carboxyl groups (553 cm^−1^ and 748 cm^−1^) [47,52], proving the successful preparation of MIL-101(Fe). Moreover, the characteristic peaks of sodium metasilica included Si-O-Si stretching vibrations (1022 cm^−1^, 707 cm^−1^ and 587 cm^−1^) [53,54], indicating that silicic acid could be adsorbed on the surface of MIL-101(Fe).

**Figure 3:**
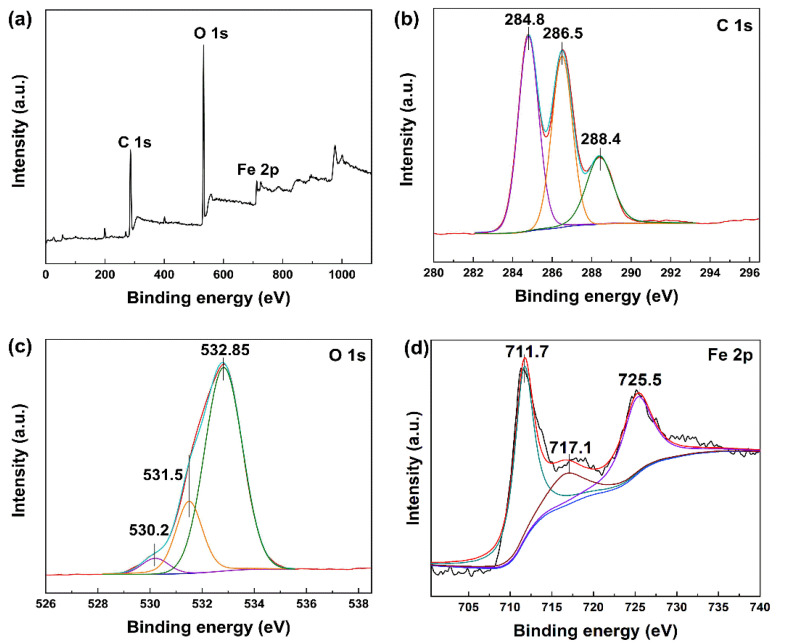
XPS spectra of MIL-101(Fe): (**a**) survey scan, (**b**) C 1s (61.7%), (**c**) O 1s (35.2%) and (**d**) Fe 2p (3.1%).

XPS spectra were further performed to analyze the surface elements and chemical bonds of MIL-101(Fe). As seen from Figure 3, the C 1s peak could be divided into three peaks at 284.8 eV, 286.5 eV and 288.4 eV, which were attributed to C-C, C-O and C=O bonds, respectively. In addition, the peaks at 530.2 eV, 725.5 eV and 711.7 eV could be assigned to Fe-O bonds, Fe 2p_1/2_ and Fe 2p_3/2_, respectively. The Fe 2p_1/2_ and Fe 2p_3/2_ peaks with a satellite signal at 717.1 eV indicated the presence of Fe^3+^ within MIL-101(Fe) [55]. Peaks at 709.0 eV and 722.6 eV were not observed, suggesting that Fe^2+^ was not incorporated within MIL-101(Fe).

### 3.3. Silicic Acid Adsorption of MIL-101(Fe)

#### 3.3.1. Effect of Initial pH on Silicic Acid Adsorption

**Figure 4:**
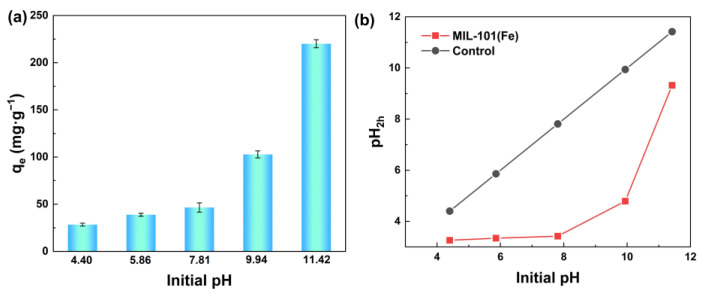
(**a**) Effect of initial pH value on the adsorption performance of silicic acid; (**b**) pH value changing trend with time.

Figure 4a shows that the adsorption ability of MIL-101(Fe) to silicic acid was closely related to the initial pH of the sodium metasilica solution. Sodium metasilica mainly existed in the form of silicic acid molecules when the pH was below 9.83, resulting in a low adsorption capacity. When the pH reached 11.42, silicic acid existed in the form of ions, and the maximum adsorption capacity of MIL-101(Fe) to silicic acid reached 220.1 mgSiO_2_·g^−1^, revealing the high adsorption ability of MIL-101(Fe).

Figure 4b shows the changing trend of pH after adding MIL-101(Fe) to silicic acid solution under different pH conditions. Since both silicic acid and MIL-101(Fe) were negatively charged under alkaline conditions, the electrostatic attraction between them cannot explain the adsorption process well. Thus, we held the opinion that the high adsorption capacity of MIL-101(Fe) to silicic acid when the pH reached 9.83 might be due to the formation of Fe-OH groups, and then these groups reacted with -OH groups of silicic acid to form Fe-O-Si bonds. As the adsorption progressed continuously, the OH- in the solution was largely consumed, resulting in a significant decrease in the pH value after the adsorption.

#### 3.3.2. Effect of Mixing Time and Kinetics on Silicic Acid Adsorption

**Figure 5:**
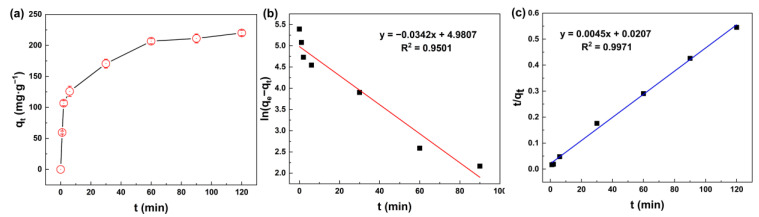
(**a**) Adsorption capacity of silicic acid as a function of time; (**b**) Linear fit using the pseudo-first-order model; (**c**) Linear fit using the pseudo-second-order model.

The effect of mixing time shown in Figure 5a exhibited the fast adsorption of silicic acid on MIL-101(Fe). At the beginning of the adsorption process, the adsorption capacity increased rapidly, and then reached an invariable value. In the first 6 min, the adsorption capacity reached 125.9 mgSiO_2_·g^−1^, and then slowed down. The sample reached adsorption saturation at about 2 h, and the maximum adsorption capacity was 220.1 mgSiO_2_·g^−1^.

To quantify the adsorption rate, the pseudo-first-order and pseudo-second-order equations were used to describe the process of adsorption, as show in Figure 5b,c. Model parameters such as the adsorption rate constant and equilibrium capacity could be calculated according to the intercept and slope of the fitted curve, as shown in Table 1. The pseudo-first-order and pseudo-second-order equations are normally expressed as Equations (3) and (4):

(3) $$q_{t}=q_{e}\left[1-exp\left(-k_{1}t\right)\right]$$

(4) $$\frac{t}{\;q_{t}}=\frac{1}{k_{2}q_{e}^{2}}+\frac{t}{q_{e}}$$

where *q_e_* (mg·g^−1^) and *q_t_* (mg·g^−1^) are the mass of silicic acid adsorbed at equilibrium and at time *t* (min), respectively; *k*_1_ and *k*_2_ are the adsorption rate constant; and *t* (min) is the mixing time.

Table 1 shows that the coefficient of determination (R^2^) of both equations was greater than 0.99, indicating a good fit of the data. The adsorption process of MIL-101(Fe) to silicic acid was consistent with the pseudo-second-order kinetic equation due to its higher R^2^, indicating that chemisorption played a leading role during the adsorption process.

#### 3.3.3. Effect of Initial Concentration and Adsorption Isotherm Study

**Figure 6:**
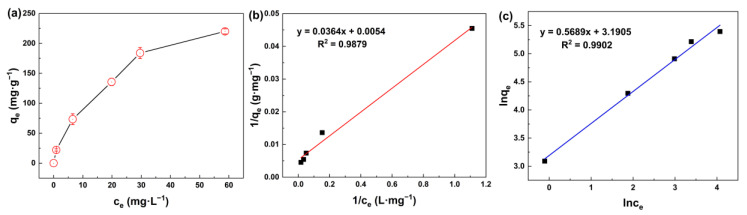
(**a**) Adsorption capacity of silicic acid as a function of initial concentration; (**b**) Adsorption isotherms of the silicic acid adsorption process with the Langmuir isotherm model; (**c**) Adsorption isotherms of the silicic acid adsorption process with the Freundlich isotherm model.

Figure 6a exhibits the effect of the initial concentration of silicic acid on the adsorption performance. With the initial concentration increasing gradually, MIL-101(Fe) showed a rapid adsorption of silicic acid at the beginning, and then the adsorption rate decreased due to the limited active sites.

Langmuir and Freundlich isotherm models [56] are normally expressed as Equations (5) and (6), respectively:

(5) $$q_{e}=\frac{q_{m}C_{e}}{\frac{1}{b}+C_{e}}$$

(6) $$q_{e}=K_{f}C_{e}^{1/n}$$

where *q_m_* (mg·g^−1^) is the maximal adsorption capacity, *C_e_* (mg·L^−1^) is the equilibrium concentration and *b* (L·mg^−1^) is the equilibrium constant in the Langmuir model. *K_f_* (mg·g^−1^) and n are the empirical coefficients in the Freundlich model.

**Figure 7:**
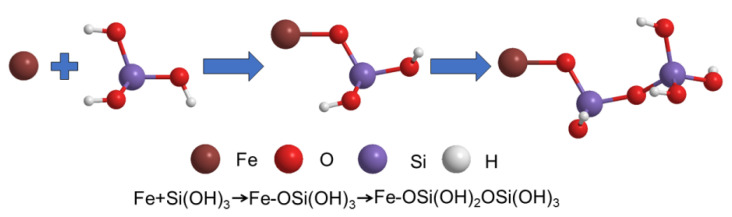
Chemical diagram involving the two-step mechanism.

In general, the Langmuir model assumes that only monomolecular layer adsorption occurs on the solid surface, which means that the adsorbate only interacts with the surface of the adsorbent, whereas the Freundlich model is applicable for multilayer adsorption, in which the adsorbates can interact with each other. It can be seen from Figure 6b,c and Table 2 that the R^2^ of both isotherm models are above 0.98, indicating that both monomolecular layer adsorption and multimolecular layer adsorption occurred during the adsorption of silicic acid on the surface of MIL-101(Fe). Thus, the adsorption process of silicic acid on MIL-101(Fe) involves two steps, as can be seen in Figure 7. First, silicic acid would bind directly to the unsaturated ion sites, which was the main mechanism of the rapid adsorption. As the adsorption proceeded, all the ion sites were occupied, resulting in the free silicic acid interacting with the adsorbed silicon to form Si-O-Si bonds.

#### 3.3.4. Effect of Temperature and Adsorption Thermodynamics Study

**Figure 8:**
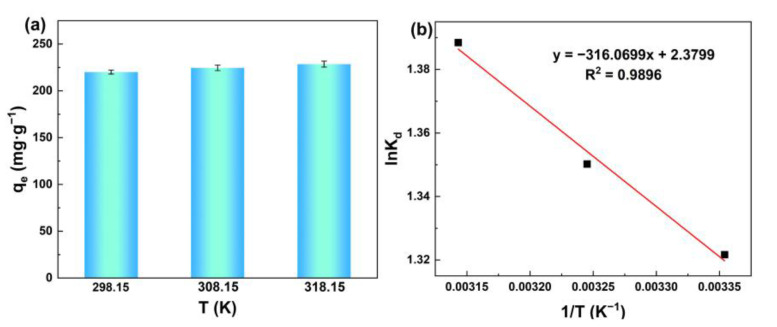
(**a**) Adsorption capacity of silicic acid as a function of the temperature; (**b**) Fitting diagram of adsorption thermodynamic model of the silicic acid adsorption process.

Figure 8a shows the effect of temperature on the adsorption of silicic acid. Obviously, the adsorption capacity to silicic acid increased with increasing temperature, indicating the benefits of heating on silicic acid adsorption.

The Van’t Hoff equation was applied to calculate thermodynamic parameters, as shown in Equations (7)–(9) [57]:

(7) $$k=\frac{q_{e}}{C_{e}}$$

(8) $$\ln k=\left(-\frac{\Delta H}{R}\right)\cdot \frac{1}{T}+\frac{\Delta S}{R}$$

(9) ΔG=−RTlnk

where *k* is the adsorption coeffcient; *T* (K) is the thermodynamic temperature; Δ*H* (kJ·mol^−1^) is the adsorption enthalpy; Δ*S* (J·mol^−1^·K^−1^) is the adsorption entropy; *R* (J·mol^−1^·K^−1^) is gas constant; and Δ*G* (kJ·mol^−1^) is the adsorption Gibbs free energy.

The fitting diagram and thermodynamic parameters can be seen in Figure 8b and Table 3. Δ*H* > 0 demonstrates that the adsorption process is endothermic, which means that the temperature rise is beneficial to accelerate the adsorption. Δ*G* < 0 indicates that the adsorption of silicic acid on MIL-101(Fe) can occur spontaneously. Δ*S* > 0 signifies that this adsorption is a process of entropy increase.

#### 3.3.5. Adsorption Mechanism Study

**Figure 9:**
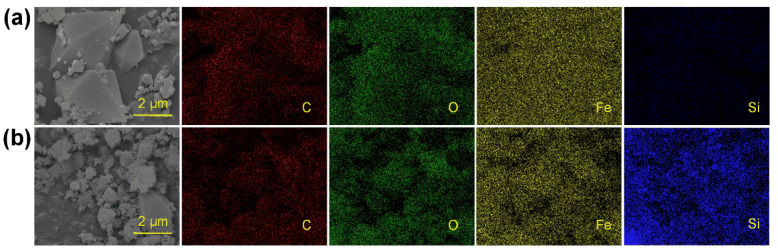
SEM image and elemental mapping of MIL-101(Fe) before (**a**) and after (**b**) adsorption of silicic acid.

Figure 9 shows the SEM images and elemental mapping of MIL-101(Fe) before and after the adsorption of silicic acid. The Si signal on the surface after the adsorption was significantly stronger than that before the adsorption, mainly due to the coverage of ion sites with silicic acid. The elemental composition of MIL-101(Fe) was also investigated using elemental mapping. Specifically, the weight concentration of oxygen on the surface increased from 22.70% to 25.11% while that of silicon increased from 0.62% to 3.34%. It can be proven using elemental composition analysis that silicic acid can not only bind to the unsaturated ion sites, but also interact with the adsorbed silicon, which was important evidence of the adsorption mechanism of silicic acid on MIL-101(Fe).

Recycle and regeneration experiments of MIL-101(Fe) were conducted immediately after the 2 h adsorption test. After washing MIL-101(Fe) with 0.05 M NaOH used as a regeneration reagent to release the silicic acid three times, ~75.6% of the adsorbent could be recycled and it showed ~60% recovery of the adsorption capacity.

#### 3.3.6. Silica-Scale Mitigation on RO Membranes

**Figure 10:**
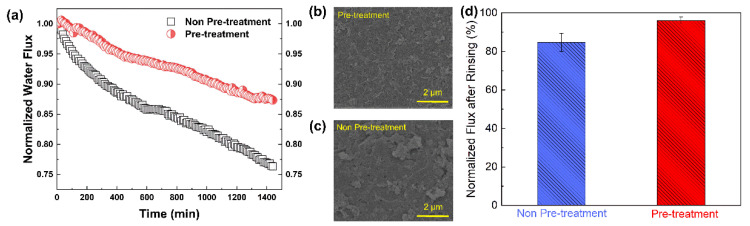
(**a**) Normalized water flux as a function of filtration time for an RO membrane without pretreatment or with pre-treatment of the feed solution with MIL-101(Fe); (**b**,**c**) Surface SEM images of the RO membrane after filtration; (**d**) Normalized flux after chemical cleaning of the membranes that experienced the silica-scaling experiment.

To investigate the potential application of MIL-101(Fe) for silica-scaling mitigation in RO systems, a control experiment was designed. With other conditions being the same, the feed solution was pretreated with MIL-101(Fe) to adsorb silicic acid. As shown in Figure 10a, when the feed solution was not pretreated, the RO membrane showed a flux decline of around 23% after 24 h continuous filtration. Comparatively, when the feed solution was pretreated by the adsorption of MIL-101(Fe), the membrane flux only decreased by 13% after 24 h continuous filtration. Therefore, it can be concluded that if the feed solution is pretreated, less silica scale will form during the RO process, mainly because a large amount of silicic acid in the feed solution is adsorbed by MIL-101(Fe). Moreover, the membrane surface after filtration was observed by SEM, as presented in Figure 10b. In the non-pretreatment group, silica scale was concentrated on the membrane surface, while in the MIL-101(Fe) adsorption pretreatment group, there was a visible peak–valley structure and less scale on the RO membrane surface.

Moreover, after 24 h of silica scaling, the membrane was cleaned with 0.1 M NaOH [18] with a flow rate of 1.2 L/min. After 2 h of cleaning, deionized water was used to rinse the membrane and filtration system. The membrane flux recovery rate was calculated using the initial flux and the flux after cleaning. As shown in Figure 10d, the water flux recovery rates of the membranes reached 96.1% for the pretreatment group and 84.7% for the non-pretreatment group, suggesting that chemical cleaning using an NaOH solution could effectively restore water flux by increasing silica solubility and depolymerizing colloidal silica [23]. In addition, the adsorption pretreatment of the feed solution favored an improvement in the efficiency of chemical cleaning, since the polymerization degree of silicic acid was greatly reduced after the adsorption [29]. Thus, the pretreatment of the feed solution based on the adsorption of MIL-101(Fe) became a more efficient approach for mitigating silica scale on RO membranes.

## 4. Conclusions

In this work, the metal-organic framework MIL-101(Fe) was successfully synthesized for the adsorption of silicic acid. Owing to its numerous unsaturated ion sites, MIL-101(Fe) exhibited excellent adsorption ability for silicic acid. The results of the adsorption experiment showed that the maximum adsorption capacity of MIL-101(Fe) for silicic acid could reach 220.1 mgSiO_2_·g^−1^. On account of the positive correlation between OH^−^ and adsorbed silicic acid, the adsorption process of silicic acid on MIL-101(Fe) should involve two steps. Firstly, silicic acid binds directly to the unsaturated ion sites. As the adsorption proceeds, all the ion sites are occupied, resulting in the free silicic acid interacting with the adsorbed silicon to form Si-O-Si bonds. When the feed solution of RO systems was pretreated with the adsorption of MIL-101(Fe), the membrane flux only decreased by 13% after 24 h of continuous filtration, indicating that the adsorption treatment with MIL-101(Fe) could effectively be applied for silica-scaling mitigation in RO systems.

## Figures and Tables

**Table 1:** Kinetic parameters of silicic acid adsorption process.

Adsorbent	q_e_ (mg·g^−1^)	Pseudo-First-Order			Pseudo-Second-Order		
		k_1_	q_e,c_ (mg·g^−1^)	R^2^	k_2_	q_e,c_ (mg·g^−1^)	R^2^
MIL-101(Fe)	220.1	0.0342	13.54	0.9501	9.78 × 10^−4^	222.22	0.9971

**Table 2:** Isotherm parameters of silicic acid adsorption process.

Adsorbent	Langmuir Model			Freundlich Model		
	q_m_ (mg·g^−1^)	b	R^2^	K_f_ (mg·g^−1^)	n	R^2^
MIL-101(Fe)	185.18	0.1484	0.9879	8.6727	1.7578	0.9902

**Table 3:** Thermodynamic parameters for silicic acid adsorption on MIL-101(Fe).

*T* (K)	Δ*G* (kJ·mol^−1^)	Δ*H* (kJ·mol^−1^)	Δ*S* (J·mol^−1^·K^−1^)
298.15	−3.276	2.628	19.786
308.15	−3.459		
318.15	−3.673		

## Data Availability

Not applicable.
